# Half-metallicity of the (001), (111) and (110) surfaces of CoRuMnSi and interface half-metallicity of CoRuMnSi/CdS

**DOI:** 10.1039/c8ra02918k

**Published:** 2018-07-18

**Authors:** Jabbar M. Khalaf Al-zyadi, Ammar A. Kadhim, Kai-Lun Yao

**Affiliations:** Department of Physics, College of Education for Pure Sciences, University of Basrah Iraq Jabbar_alzyadi@yahoo.com +94-77-07342323; School of Physics and Wuhan National High Magnetic Field Center, Huazhong University of Science and Technology Wuhan 430074 China; International Center of Materials Physics, Chinese Academy of Sciences Shenyang 110015 China

## Abstract

Recent studies have indicated that the quaternary Heusler alloy CoRuMnSi shows a half-metallic ferromagnetism (Kundu *et al.*, *Sci. Rep.*, 7, (2017), 1803). The (111), (110), and (001) surfaces and the interfaces with CdS (111) substrate of the quaternary Heusler alloy CoRuMnSi were explored by carrying out a first-principles investigation based on a density functional theory. Calculations showed that the half metallicity can be preserved for the Si-terminated (111) surface and subsurface while the half-metallicity approved in the bulk CoRuMnSi is destroyed at Co, Ru, and Mn-terminations (111) surfaces and subsurfaces. Regrettably, the surface states ruin the gap in the spin-down channel at both MnSi- and CoRu-terminated (001) surfaces and subsurfaces. Remarkably, the (110) surfaces and subsurfaces have a nearly half-metallicity property with a high spin polarization. Based on spin magnetic character calculations, the spin magnetic moments of surface and subsurface atoms are larger and smaller than those in the bulk quaternary Heusler alloy CoRuMnSi. For the interface of CoRuMnSi/CdS (111), the bulk half-metallicity is destroyed at Si–Cd and Si–S configurations.

## Abstract 2

Recent studies have indicated that the quaternary Heusler alloy CoRuMnSi shows a half-metallic ferromagnetism (Kundu *et al.*, *Sci. Rep.*, 7, (2017), 1803).

## Graphical abstract

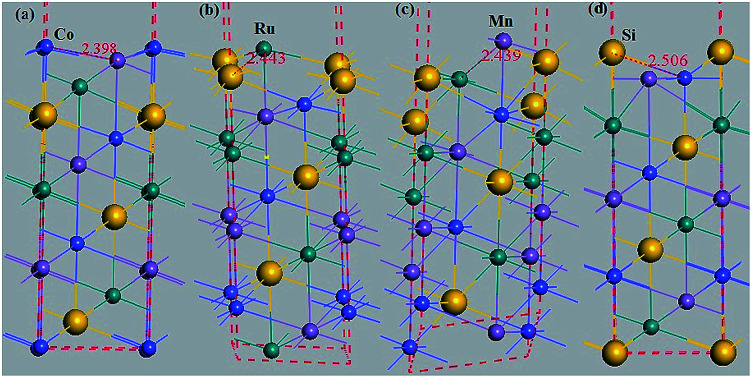

## 1. Introduction

Half-metals demonstrate complete spin-polarized band structures that fit in with spintronic applications.^1–3^ A half-metal is a material in which one of the spin states reveals a metallic behavior while the other that stands for a semiconducting behavior is called a half-metallic ferromagnet. Therefore, such types could create a cause for spin-polarized currents to occur. Theoretically, the half-metallic ferromagnetism was first accepted as a fact in NiMnSb by de Groot *et al.*^4^ Half-metallicity can be observed in various classes of materials, like magnetic oxides,^5^ diluted magnetic semiconductors,^6^ and Heusler alloys.^7^ A large number of intermetallic compounds of the type X_2_YZ have been discovered since the discovery of the first Heusler compounds, Cu_2_Mn (Al, Sn, Zn, Bi, Sb, or B), by Heusler in 1903.^8^ Typically, the X and Y are considered transition elements while the Z belongs to Group III–V in the periodic table. Much attention has been directed to the Heusler alloys owing to their high Curie temperatures and tunable electronic structures. However, not all compounds of this stoichiometry contain the original prototype Cu_2_MnAl and a space group (*Fm* 3*m*) structure. Accordingly, there are practically thousands of such compounds. After their discovery, and for decades, these intermetallics were little more than a scientific curiosity studied generally due to their ferromagnetic properties. But, a growing interest in other functional properties has been observed in the last few years.^9^ These involve magnetic shape memory, magneto-caloric effects, thermoelectric properties, and spintronic behavior amongst others.^10–13^ In a Heusler compound, the required condition for a half-metallic behavior is that the total magnetic moment per formula unit is an integer and that it follows and adopts the Slater–Pauling rule,^14–16^ which links the total magnetic moment to the number of valence electrons. According to Heusler alloys, the magnetization *M* and the number of valence electrons *N*_ν_ are linked or connected either by *M* = *N*_ν_ − 18 or by *M* = *N*_ν_ − 24.^17^ The half-metallic quaternary Heusler compounds were found to obey these rules. It was proved that the cobalt-based Heusler alloys are very interesting because of their theoretically predicted half metallic electronic structures and that they experimentally exhibit a high spin polarization and high Curie temperatures.^18–24^ The half-metallic property and structure in a quaternary Heusler alloy CoRhMnSi were theoretically determined by Kundu *et al.*^25^ It is very significant to clearly inspect and examine the surface properties and their interface with semiconductors for practical spintronic implementations due to the fact that the surface and interface usually produce an effect and even damage the half-metallicity of the bulk.^26–30^

In this paper, former studies on a HM ferromagnet of the quaternary Heusler compound CoRhMnSi^25^ to the CoRuMnSi (111), (001), and (110) surfaces and the CoRuMnSi/CdS (111) interface have been expanded by employing first-principles calculations. The results demonstrate that the bulk HM property is maintained only at the Si-terminated (111) surface. The two possible configurations of the CoRuMnSi/CdS (111) interface unluckily damage and ruin the HM characteristic.

## 2. Computational method

In order to investigate the structural, electronic, and magnetic properties of the CoRuMnSi compound, first-principles calculations which employ the pseudo-potential plane-wave method^31^ with the Wien2k package^32^ were performed. We chose the exchange-functional correlation using the generalized gradient approximation in the scheme of Perdew–Burke–Ernzerhof (PBE).^33^ The 12 × 12 ×1 *k* meshes for the (111), (001) surfaces, and interfaces with semiconductors, and the 9 × 12 × 1 *k* meshes for (110) surface were selected. As for the comparison with the surfaces and interface, 12 × 12 × 12 *k* meshes were employed to calculate the bulk quaternary Heusler alloy CoRhMnSi and zinc-blende (ZB) CdS. In the study of the bulk CoRhMnSi, the (111), (001), and (110) surfaces and the CoRuMnSi/CdS (111) interface, the radii *R*_mt_ of the muffin tins are considered to be 2.3 a.u. for all the Co, Rh, Mn, Cd, S, and Si atoms. We used *R*_mt_ × *K*_max_ equal to 8.5 and made the angular momentum expansion up to *l* = 10 in the muffin tins. When the total energy disparity between succeeding iterations was less than 10^−5^ Ry per formula unit, this meant that the self-consistency calculations were achieved. The crystal structure of the quaternary Heusler alloy CoNbMnSi is known to be a highly ordered Hg_2_CuTi-type structure. The type of atom arrangement in the quaternary Heusler compound is X_1_X_2_YZ: X_1_ (1/4, 1/4, 1/4), X_2_ (3/4, 3/4, 3/4), Y (1/2, 1/2, 1/2), and Z (0, 0, 0).^25^

## 3. Results and discussion

### 3.1 Surface properties

The equilibrium lattice constant adopted to construct the (111), (001), and (110) surfaces for the quaternary Heusler alloy CoRuMnSi is the previous calculated value of 5.79 Å.^25^

For practical applications in spin electronic devices, the HM materials are usually simulated to a surface and interface. Nevertheless, the electronic properties of a surface commonly vary from those of the bulk. The surface and interface are able to reduce the spin-polarization values and destroy the half-metallicity. So, augmentation of the surface and the interface effects on half-metallicity are very significant. The (111) surface has four types of terminations: Co-, Ru-, Mn-, and Si-. For the Co-terminated (111) surface, the slab contains four Co, three Ru, Mn and Se atoms layers, but the slabs of (Ru- and Mn-) Si-terminations have four (Ru and Mn) Si, three (Co and Co) Co, three (Mn and Ru) Ru and three (Si and Si) Mn atoms layers. In the case of the (110) surface, there is just one kind of termination with Co, Ru, Mn, and Si, a situation that indicates that each layer contains Co, Ru, Mn, and Si together (where the slab comprises seven Co, Ru, Mn, and Si atoms layers for the surface).

**Fig. 1:**
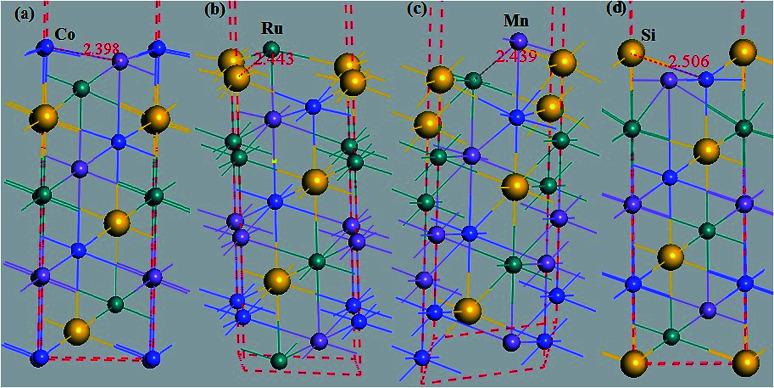
Slab models used to study the quaternary Heusler alloy CoRuMnSi (111) surfaces. (a) The Co-terminated (111) surface, (b) Ru-terminated (111) surface, (c) Mn-terminated (111) surface, and (d) Si-terminated (111) surface, where the bond lengths are indicated.

**Fig. 2:**
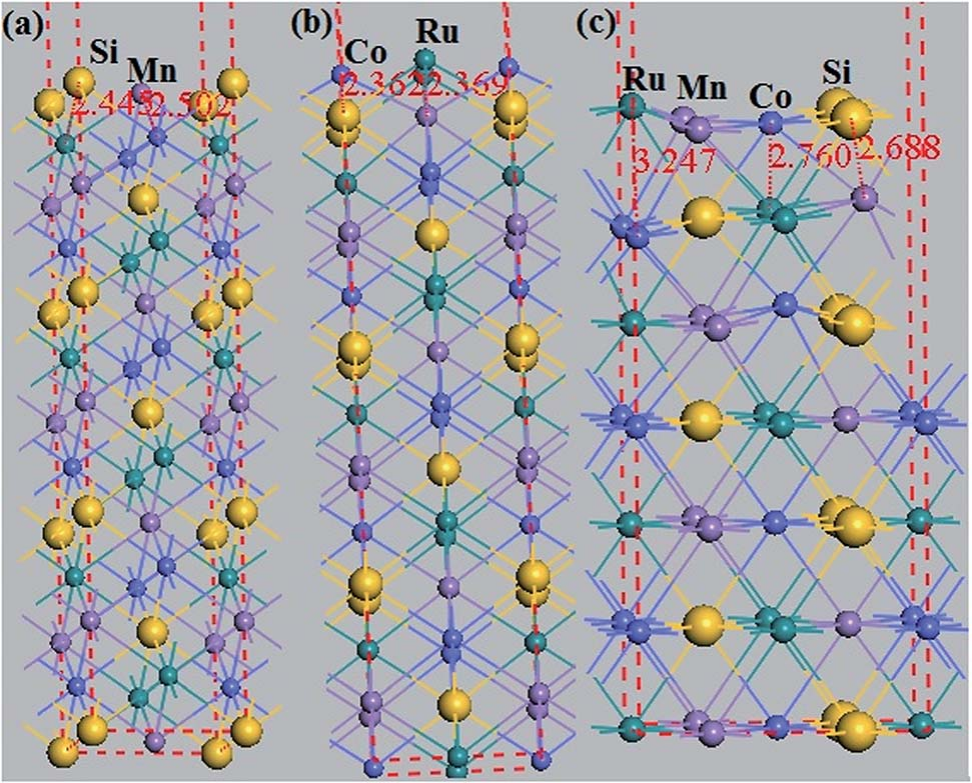
(a) The thirteen-layer MnSi-terminated (001) surface and (b) CoRu-terminated (001) surface for the CoRuMnSi compound. (c) The seven-layer CoRuMnSi-terminated (110) surface for the CoRuMnSi compound.

**Fig. 3:**
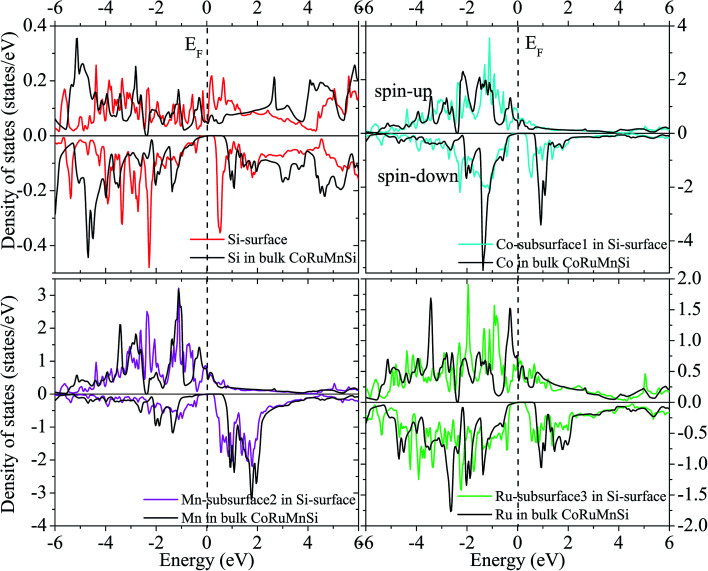
Spin-dependent DOS of Si atom at the Si-terminated (111) surface of CoRuMnSi, and Co, Mn, and Ru atoms at the subsurfaces. For comparison, the DOS of Si, Co, Mn, and Ru atoms in bulk CoRuMnSi are also provided. The dashed line exhibits the Fermi level at 0 eV.

**Fig. 4:**
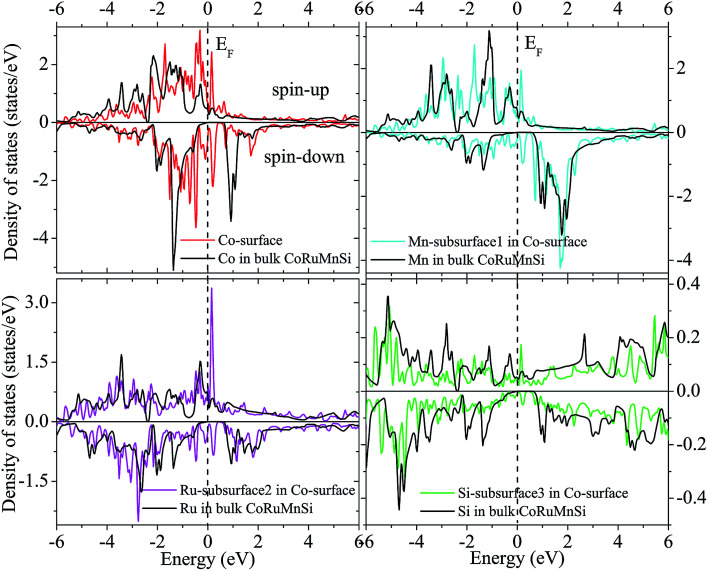
Similar to Fig. 3 for the case of Co-terminated (111) surface of CoRuMnSi.

**Fig. 5:**
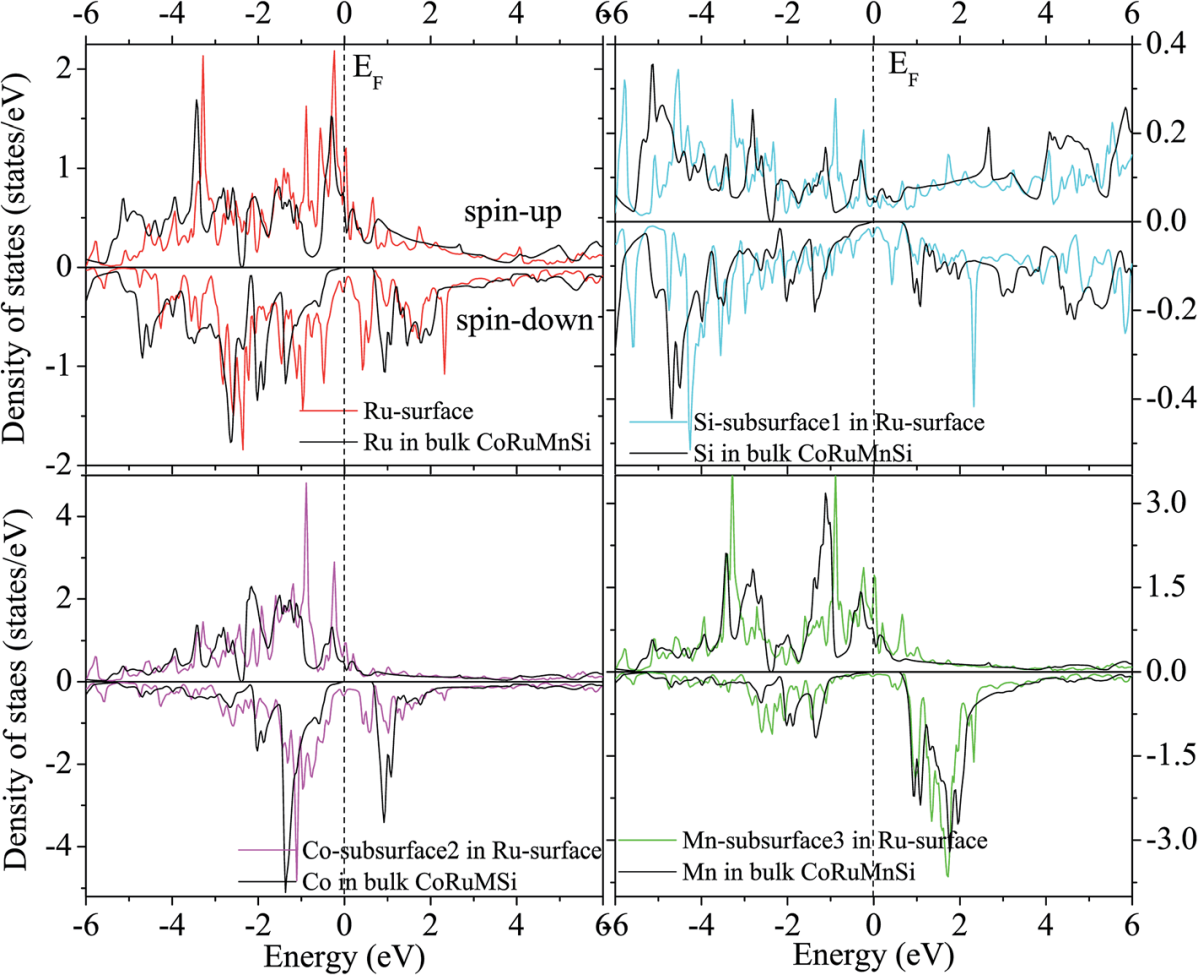
Similar to Fig. 3 for the case of Ru-terminated (111) surface of CoRuMnSi.

**Fig. 6:**
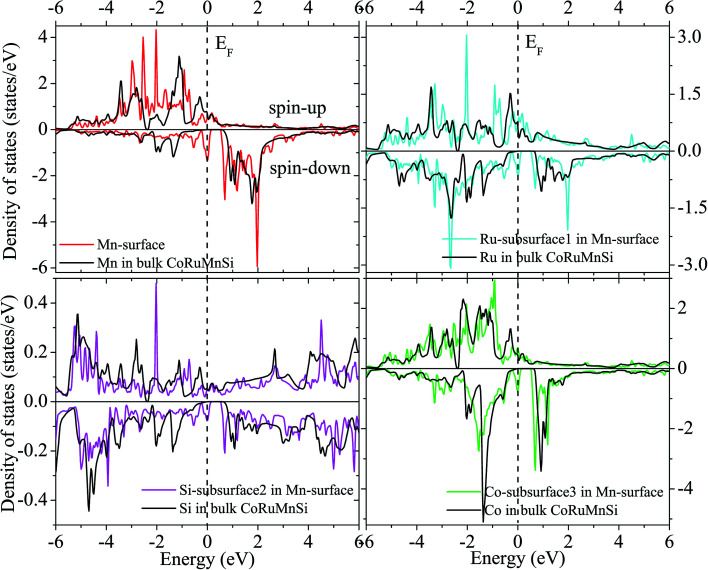
Similar to Fig. 3 for the case of Mn-terminated (111) surface of CoRuMnSi.

Additionally, the (001) surface of a CoRuMnSi Heusler alloy comprises alternating atomic layers which contain only one of the Mn and Si or Co and Ru atoms. Therefore, there are two types of terminations for a (001) surface, which are named as MnSi- and CoRu-terminated (001) surfaces. The slab contains thirteen monolayers of atoms and a 15 Å vacuum above the (111) and (001) surfaces to avert the interactions of consecutive slabs. However, a slab of seven-layers proved to be adequate for studying the (110) surface. To shape epitaxial (111) and (001) surfaces, the topmost four atomic layers are allowed to relax, and the internal nine atomic layers are fixed as slabs by minimizing the total energy and the atomic forces (see Fig. 1 and 2a and b). Therefore, we obtain the equilibrium surface structure for both surfaces. Moreover, we obtain the equilibrium structure of the (110) surface of CoRuMnSi by allowing the top three atomic layers to relax and the internal four atomic layers are fixed (see Fig. 2c). The number of relaxed and slab layers is enough to study the surface characteristics. The differences of spin magnetic moment and bond length values of the surfaces are very impalpable when vacuums and thicker slabs are employed. The changes are less than 0.0015 *μ*_B_ for the spin magnetic moment value, and less than 0.003 Å for the bond length of surface atoms. Based on the above, we studied the electronic structure, magnetic properties, spin-polarization, half-metallicity, and base on the terminated (111), (001), and (110) surfaces for the CoRuMnSi compound. We perceive that the distance between the subsurface and surface atoms, is 2.507 Å for the Co-, Ru-, Mn-, and Si-terminated (111) surfaces before relaxation, while after relaxation, it becomes 2.506, 2.443, 2.439, and 2.398 for the Si-, Ru-, Mn-, and Si-terminated (111) surfaces, respectively. The bond lengths are particularly reduced by 0.11, 0.068, 0.064, and 0.001 Å for the Co-, Mn-, Ru-, and Si-terminated (111) surfaces of CoRuMnSi, respectively. In the case of the CoRu-terminated (001) surface, before relaxation the bond lengths are 2.507 Å. After relaxation, the Co–Mn, Co–Si, Ru–Mn, and Ru–Si bonds lengths are 2.321, 2.362, 2.369, and 2.412 Å, respectively. In contrast, after relaxation, the Mn–Co, Mn–Ru, Si–Co, and Si–Ru bonds lengths at the MnSi-terminated (001) surface are 2.502, 2.457, 2.489, and 2.445 Å, respectively. Fig. 3–6 show the calculated density of states (DOS) at the surface and subsurfaces of the (111) slab. These results can be illustrated by Si-, Ru-, Mn- and Si-terminated (111) surfaces. Meantime, the corresponding spin-polarized atomic DOS in the bulk was also illustrated to compare between the surfaces and the bulk system. It is clear that the Si-terminated (111) surface and subsurfaces maintain the bulk half metallic property, because the spin-down state denotes a semiconducting nature, while the spin-up state indicates a metallic demeanor. There are energy gaps in the spin-down channel of about 0.32, 0.28, 0.31, and 0.43 eV at the Fermi level for Si surface, Co subsurface (1), Mn subsurface (2), and Ru subsurface (3), respectively. For comparison, the energy gaps of the bulk are about 0.36, 0.65, 0.68, and 0.72 eV for Si, Co, Mn, and Ru, respectively. Interestingly, the energy gaps at the Co subsurface (1), Mn subsurface (2), and Ru subsurface (3) decrease greatly compared to the bulk value because the DOS below the Fermi level is separated and moved to the low energy region. However, the energy gap at the Si surface changes very slightly for which the DOS does not displace or move to the low and high energy regions as indicated in Fig. 3. Unluckily, the Co-, Ru- and Mn-terminated (111) surfaces are located down at the Fermi levels. Interestingly, the DOS is moved to the high energy region. Therefore, the bulk energy gap moves towards high energy and far away from the Fermi level, and the semiconductor property is transformed to a metal property at the spin-down.

**Fig. 7:**
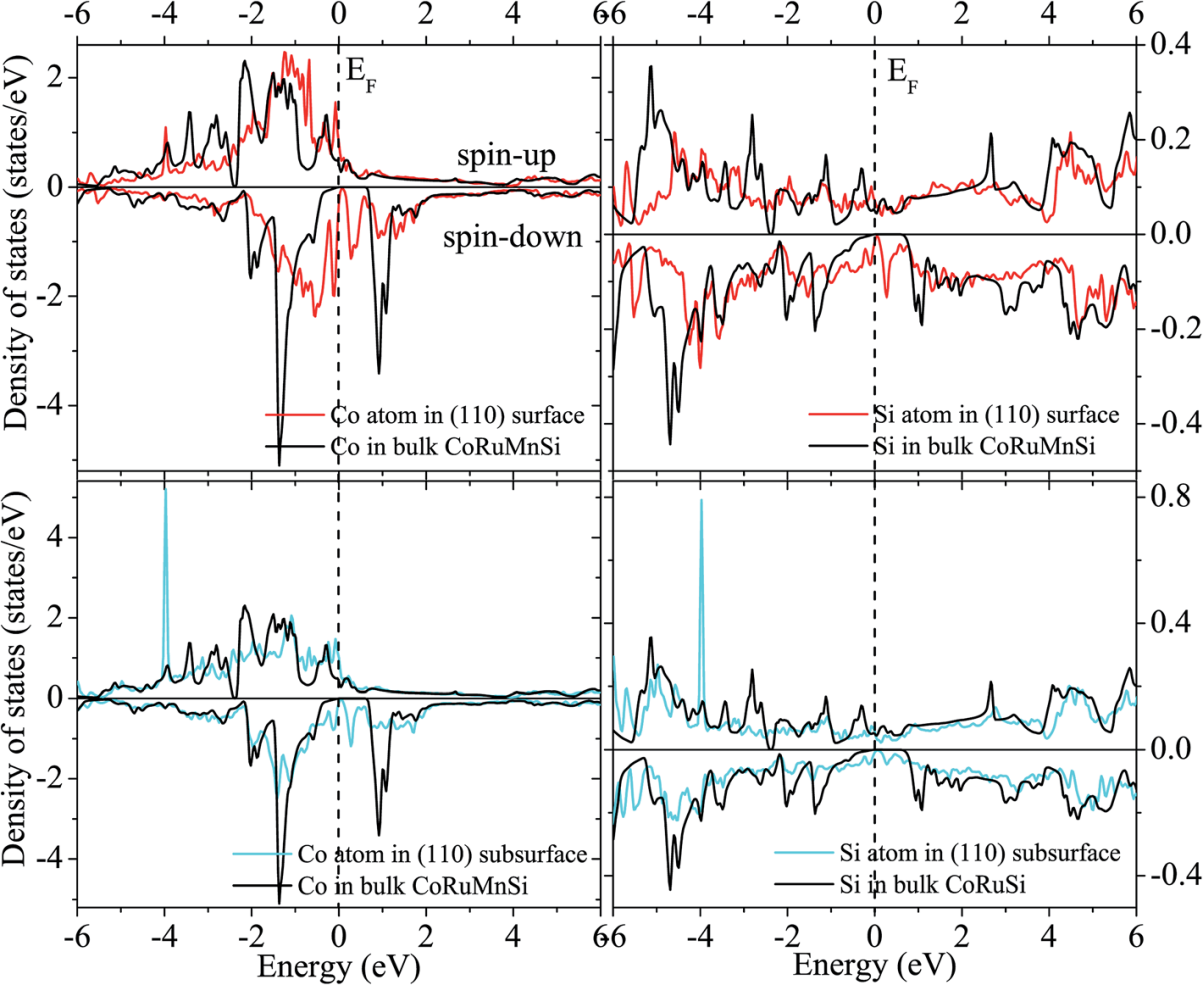
Surface and subsurface DOS of the CoRuMnSi (110) slab terminated with CoSi (red line). For comparison, the atomic-resolved DOS of Co and Si atoms in bulk CoRuMnSi are also shown (black line).

**Fig. 8:**
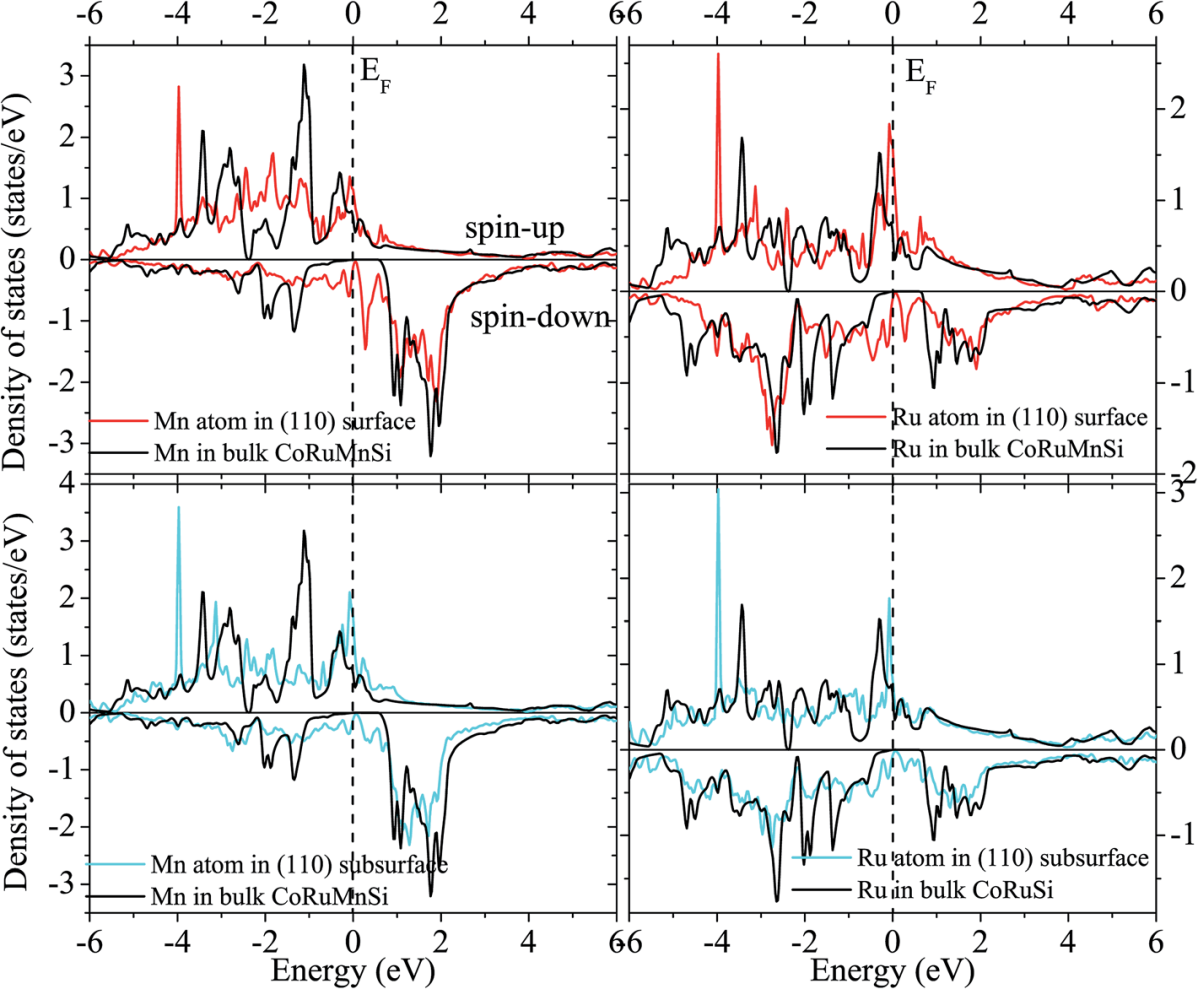
Similar to Fig. 7 exhibiting the case of the CoRuMnSi (110) slab termination with MnRu.

Uncommonly, Mn and Ru atoms at the (110) surface and subsurface as well as the up- and down-spin have high spin polarizations. Therefore, the surfaces and subsurfaces have a nearly half-metallicity properties as shown in Fig. 7 and 8. Contrarily, the Co and Si atoms have low and high spin polarizations at their surfaces and subsurfaces, respectively.

**Fig. 9:**
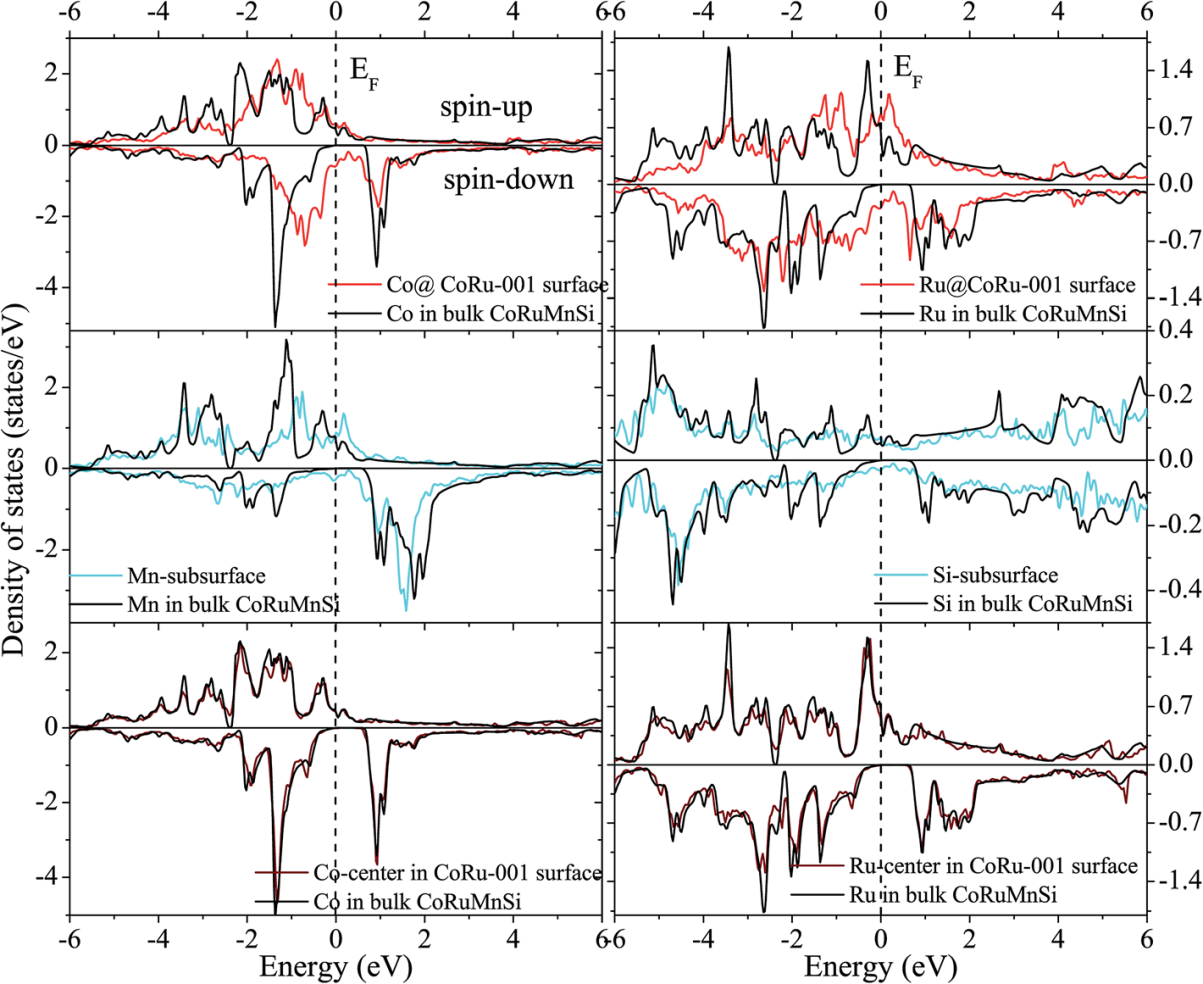
Surface, subsurface, and middle-layers DOS of the CoRuMnSi (001) slab terminated with CoRu (red line). For comparison, the atomic-resolved DOS of Co, Ru, Mn, and Si atoms in bulk CoRuMnSi are also offered (black line).

**Fig. 10:**
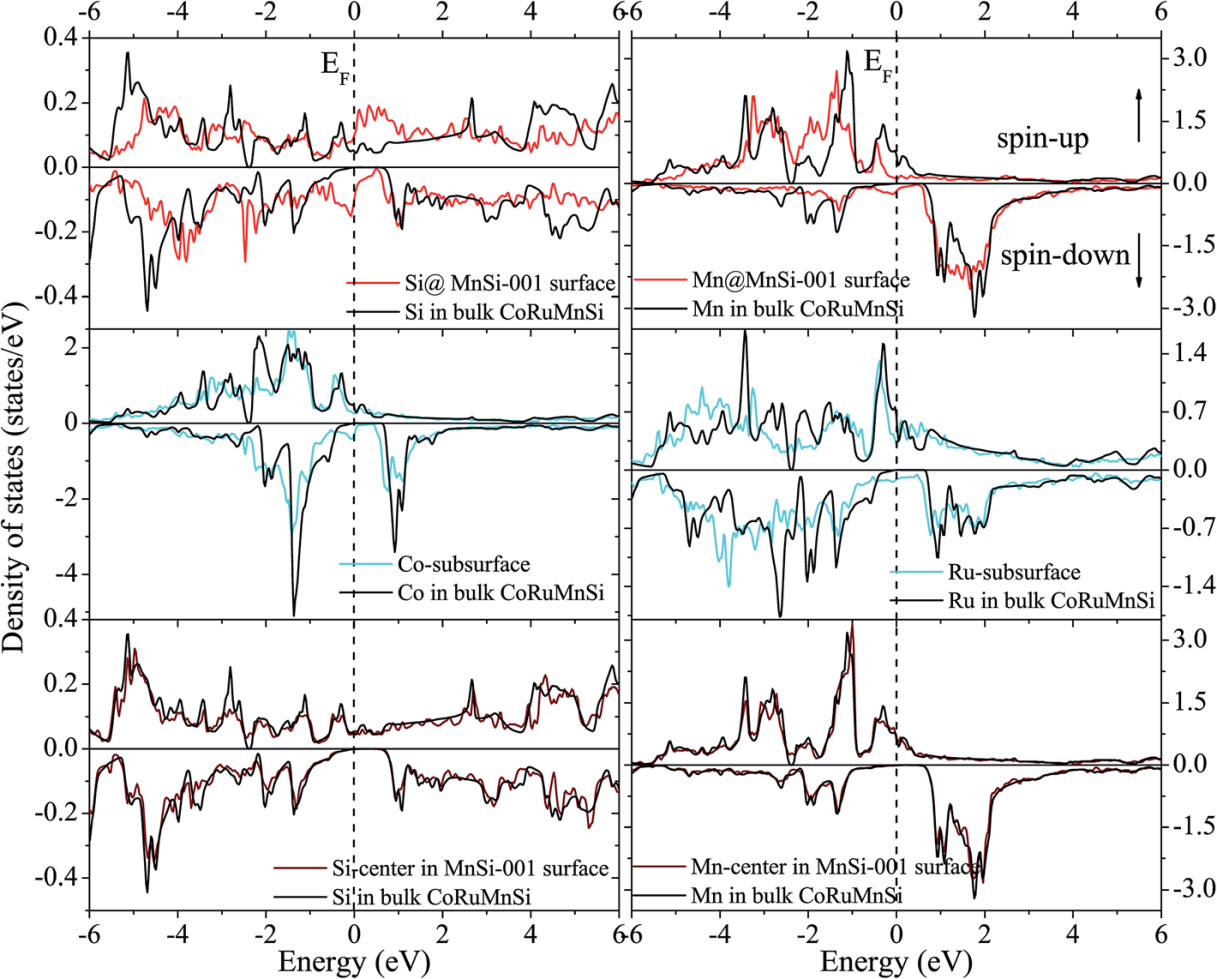
Similar to Fig. 9 exhibiting the case of the CoRuMnSi (001) slab termination with MnSi.

In contrast, surface DOS explains that the MnSi- and CoRu-terminated (001) surfaces lose the half-metallicity of the bulk of the quaternary Heusler compound CoRuMnSi. It is evident from Fig. 9 and 10 that both the majority- and minority-spin states display a metallic property at their surfaces and subsurfaces. That means that the half-metallicity of the bulk quaternary Heusler CoRuMnSi is destroyed at the MnSi- and CoRu-terminated (001) surfaces because of the occurrence of surface states in the spin-down channels. In addition, for comparison, DOS at the middle-layer of the slabs and in the CoRuMnSi bulk are given. Fig. 9 and 10 prove that for two slabs, terminated with MnSi- and CoRu-, the middle-layer atoms have DOS that are nearly the same as those in the CoRuMnSi bulk; in other words, the physical characteristics of bulk are recovered in the center-layer of the two slabs. Therefore, slabs with 13 atomic layers are convenient to investigate the properties of these surfaces.

The magnetic moments and spin polarization *P* = (*N*↑ − *N*↓)/(*N*↑ + *N*↓) at the surfaces, subsurfaces and center layers for (111), (001), and (110) surfaces were also calculated and listed in Table 1. For comparison, we calculated the *l*-decomposed down- and up-spin states within muffin tin spheres and their corresponding bulk values. The high spin polarized alloy participates in the performance of spin electronic devices. Consequently, the spin polarizations that we computed for the terminated surface and subsurface structures of the quaternary Heusler compound CoRuMnSi are higher and lower and lose half-metallicity at the (111), (001), and (110) surfaces. Depending on the detailed spin magnetic moments of surface and subsurface atoms, the spin magnetic moments of surface and subsurface atoms are evidently larger and smaller than those in the bulk system because the Co, Ru, Mn, and Si atoms at the surfaces and subsurfaces lose and gain the electron charge towards the vacuum. Interestingly, the Si magnetic moment at the (111) and (001) surfaces (0.05 and 0.13 *μ*_B_) increased greatly as compared to the bulk value (0.02 *μ*_B_). That means the surface Si spin magnetic moment was increased by about 250 and 750%. Meanwhile, the surface (111) Co spin magnetic moment and the surface (001) Ru magnetic moment decreased by about 65 and 53%, respectively. The reason is that in the bulk CoRuMnSi quaternary Heusler compound, the atoms have low-spin and high-spin states. Additionally, bond breakings at the (111) and (001) surfaces contribute to these results. Eventually, the environment in the bulk and its surface became completely different. Finally, the atomic spin magnetic moments of Co and Mn atoms at the (110) surfaces and subsurfaces were decreased, but the magnetic moments of Ru and Si atoms increased at the (110) surfaces.

**Table 1:** *l*-decomposed up (↑) and down-spin (↓) electrons within muffin-tin sphere and spin magnetic moments (in unit of *μ*_B_) for each atom in the bulk, surface (S), subsurface (Sub), and central (C) layers of the CoRuMnSi quaternary Heusler compound. The spin polarization values (*P*) calculated for the atoms are also exhibited

	Atom	s (↑/↓)	p (↑/↓)	d (↑/↓)	Total (↑/↓)	*M* (*μ*_B_)	*P* (%)
Bulk	Co	0.19/0.19	3.22/3.24	4.18/3.19	7.62/6.65	0.96	100
	Ru	1.14/1.14	3.12/3.13	2.98/2.83	7.27/7.12	0.14	100
	Mn	0.19/0.18	3.19/3.19	4.03/1.10	7.45/4.50	2.95	100
	Si	0.45/0.44	0.58/0.60	0.10/0.11	1.16/1.18	−0.02	100
Co-ter (111)	Co (S)	0.18/0.18	3.12/3.12	3.75/3.42	7.06/6.73	0.34	11.1
	Mn (Sub1)	0.18/0.16	3.14/3.13	3.86/1.09	7.21/4.40	2.81	38.0
	Ru (Sub2)	1.14/1.15	3.09/3.11	2.80/2.88	7.06/7.17	−0.11	40.2
	Si (Sub3)	0.34/0.33	0.40/0.41	0.07/0.07	0.81/0.82	−0.10	58.9
Ru-ter (111)	Ru (S)	1.07/1.07	2.96/2.96	2.72/2.50	6.75/6.54	0.22	71.3
	Si (Sub1)	0.42/0.41	0.49/0.49	0.07/0.08	0.99/1.00	−0.002	37.5
	Co (Sub2)	0.18/0.18	3.20/3.20	4.06/3.23	7.45/6.63	0.81	51.3
	Mn (Sub3)	0.13/0.12	3.10/3.09	3.55/1.21	6.80/4.44	2.36	92.1
Mn-ter (111)	Mn (S)	0.18/0.15	3.12/3.11	4.05/0.98	7.37/4.26	3.13	15.6
	Ru (Sub1)	1.13/1.14	3.07/3.08	2.78/2.90	6.99/7.14	−0.13	18.4
	Si (Sub2)	0.36/0.35	0.41/0.42	0.06/0.06	0.84/0.85	−0.11	86.5
	Co (Sub3)	0.17/0.17	3.19/3.20	4.09/3.14	7.47/6.53	0.96	82.9
Si-ter (111)	Si (S)	0.39/0.40	0.39/0.44	0.04/0.04	0.84/0.88	−0.05	100
	Co (Sub1)	0.18/0.81	3.17/3.19	3.98/3.24	7.35/6.62	0.72	100
	Mn (Sub2)	0.15/0.13	3.13/3.12	3.91/1.04	7.20/4.13	2.89	100
	Ru (Sub3)	1.11/1.12	3.07/3.08	2.76/2.79	6.96/7.00	−0.04	100
CoRu-ter (001)	Co (S)	0.17/0.16	3.13/3.12	4.05/3.11	7.35/6.40	0.95	13.23
	Ru (S)	1.10/1.11	3.02/3.02	2.73/2.80	6.86/6.94	−0.08	51.1
	Mn (Sub1)	0.16/0.15	3.14/3.13	3.55/1.36	6.88/4.66	2.22	54.7
	Si (Sub1)	0.41/0.41	0.51/0.52	0.09/0.10	1.02/1.03	−0.004	40.8
	Ru (C)	1.11/1.11	3.07/3.08	2.77/2.72	6.96/6.92	0.04	99
	Co (C)	0.15/0.15	3.16/3.17	3.98/3.16	7.30/6.49	0.81	98
MnSi-ter (001)	Si (S)	0.45/0.46	0.45/0.57	0.05/0.05	0.97/1.09	−0.13	13.0
	Mn (S)	0.16/0.13	3.10/3.09	4.28/0.71	7.56/3.93	3.62	23.1
	Co (Sub1)	0.17/0.18	3.19/3.21	4.13/3.14	7.52/6.55	0.96	16.9
	Ru (Sub1)	0.13/0.14	3.10/3.11	2.95/2.76	7.22/7.03	0.18	45.8
	Mn (C)	0.17/0.16	3.17/3.17	3.89/1.16	7.27/4.51	2.75	98
	Si (C)	0.43/0.42	0.55/0.56	0.10/0.11	1.10/1.12	−0.015	99
CoRuMnSi-(110)	Ru (S)	1.08/1.09	2.98/2.98	2.49/2.70	6.57/6.78	−0.18	90.4
	Mn (S)	0.13/0.11	3.08/3.07	3.72/1.04	6.95/4.24	2.72	81.3
	Co (S)	0.17/0.17	3.15/3.16	4.00/3.22	7.34/6.57	0.79	48.3
	Si (S)	0.40/0.40	0.43/0.45	0.51/0.05	0.89/0.92	−0.03	65
	Mn (Sub)	0.13/0.12	3.10/3.10	3.48/1.31	6.73/4.55	2.21	87.3
	Ru (Sub)	1.07/1.08	3.01/3.02	2.57/2.57	6.67/6.68	−0.006	85.2
	Si (Sub)	0.38/0.38	0.46/0.49	0.08/0.08	0.94/0.96	−0.03	74
	Co (Sub)	0.18/0.19	3.20/3.20	3.99/3.28	7.39/6.69	0.72	82

### 3.2 Interface half-metallicity

**Fig. 11:**
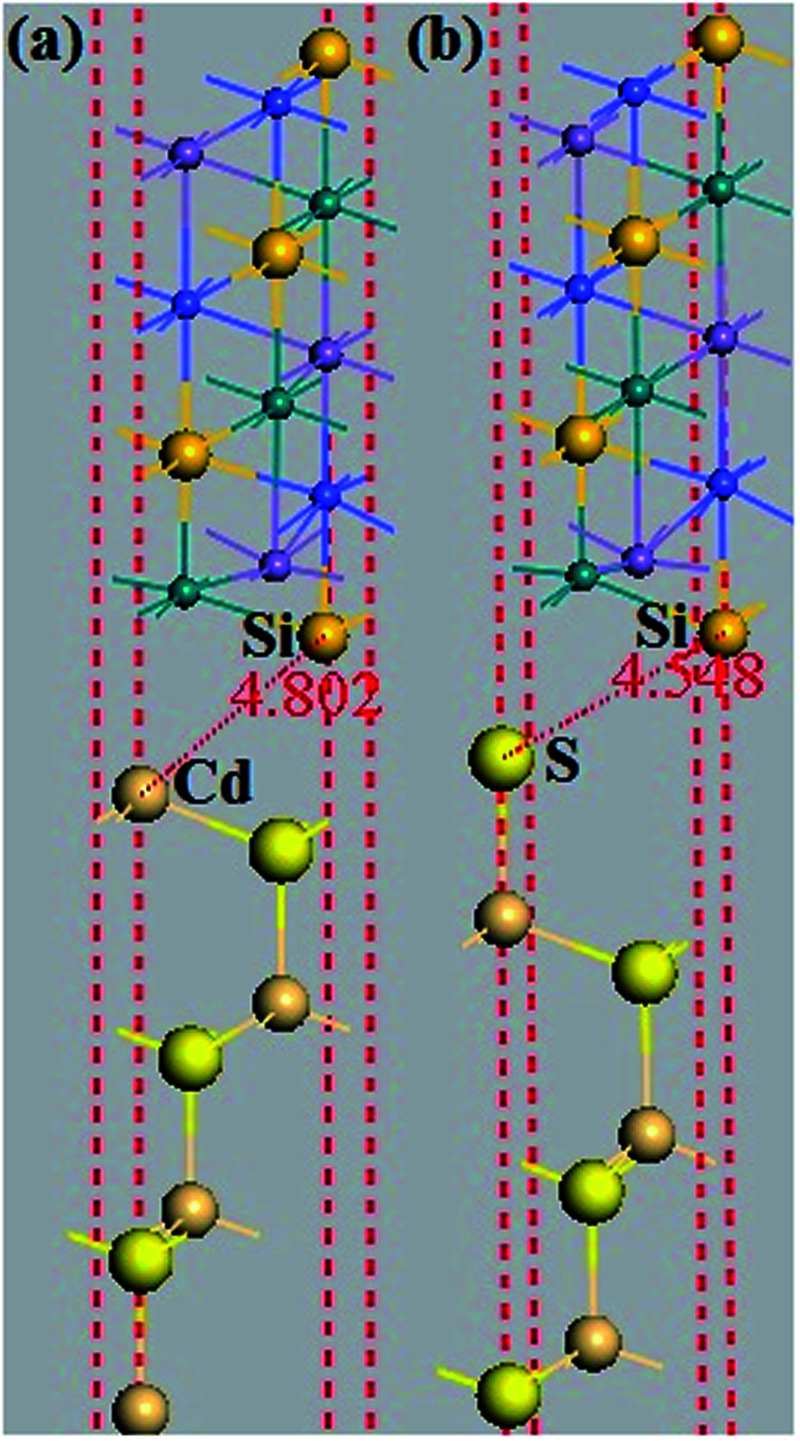
The slab models used to study the CoRuMnSi/CdS (111) interfaces. (a) The Si–Cd (111) interface, and (b) Si–S (111) interface.

It is also important to study the interface properties between the half-metal and semiconductor. Similar structures were considered to present exactly the same practical situation since in most equipment, half-metals are used to inject current into a semiconductor. We imposed that the stacking orientation was (111) and employed a slab with 13 layers of the half-metal and seven layers of the semiconductor so as to have two equivalent interfaces (see Fig. 11). Furthermore, we investigate a semiconductor that crystallizes in the ZB type. This type is suitable for the lattice structure of the quaternary Heusler alloy and in a lattice constant close to the 5.79 Å,^25^ which is the equilibrium lattice parameter of CoRuMnSi. Such a semiconductor is the binary ZB CdS. The lattice constant of ZB CdS (5.832 Å)^34^ suits well that of the quaternary Heusler alloy CoRuMnSi (5.79 Å). For the CoRuMnSi/CdS (111) interfaces, we studied only the Si-terminated (111) surface, which conserves the half-metallicity. Moreover, for CdS both Cd- and S-terminated (111) surfaces were studied. So, there are two possible terminations in CoRuMnSi/CdS (111): (1) the Si–Cd shape which means that the CoRuMnSi (111) surface is terminated at Si, and the CdS (111) surface which is terminated at Cd. Similarly, the other interfacial configuration is (2) Si–S shapes. We first performed structural relaxations for the two different configurations of the CoRuMnSi/CdS (111) interfaces. Next, the optimized bond distances between the interfacial atoms are enumerated in Table 2.

**Table 2:** The optimized bond distances (*d*_int_) between the interfacial atoms and the calculated adhesion energies for two different interfacial configurations studied here

Shape	*d*_int_ (Å)	*γ* (Jm^−2^)
Si–Cd	4.80	0.473
Si–S	4.55	0.957

The relaxed adhesion energy (111) at interfaces of Si–Cd and Si–S was considered because it is a main coefficient used to evaluate interface stability. In order to determine the stability of the interfaces of configurations, we first calculated their adhesion energy (*γ*) which can be shown as:*γ* = (*E*_CoRuMnSi_ + *E*_CdS_ − *E*_CoRuMnSi/CdS_)/*A*where *E*_CoRuMnSi_, *E*_CdS_, and *E*_CoRuMnSi/CdS_ are the total energies of the CoRuMnSi, CdS and CoRuMnSi/CdS slabs, respectively, and the interfacial area is A. We calculated their interfacial adhesion energies to compare the stability of the two different interfacial structures of the relaxed (111) interface of CoRuMnSe with CdS. The energies of adhesion values were 0.473 Jm^−2^ for Si–Cd and 0.957 Jm^−2^ for the Si–S interfacial configurations as listed in Table 2, which means that the Si–Cd shape has the smallest adhesion energy.

**Fig. 12:**
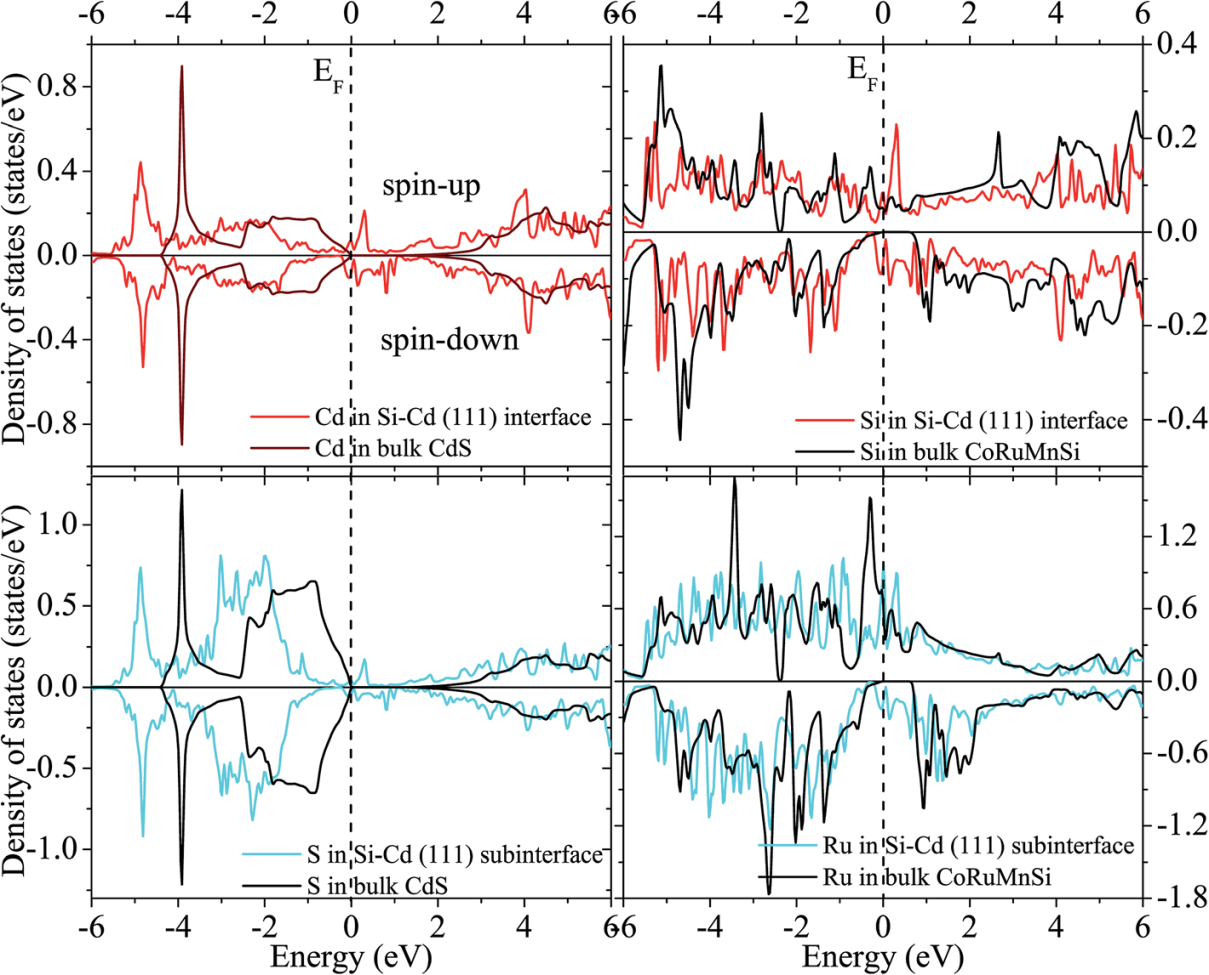
Total DOS of the interface and subinterface atoms for the Si–Cd configuration.

**Fig. 13:**
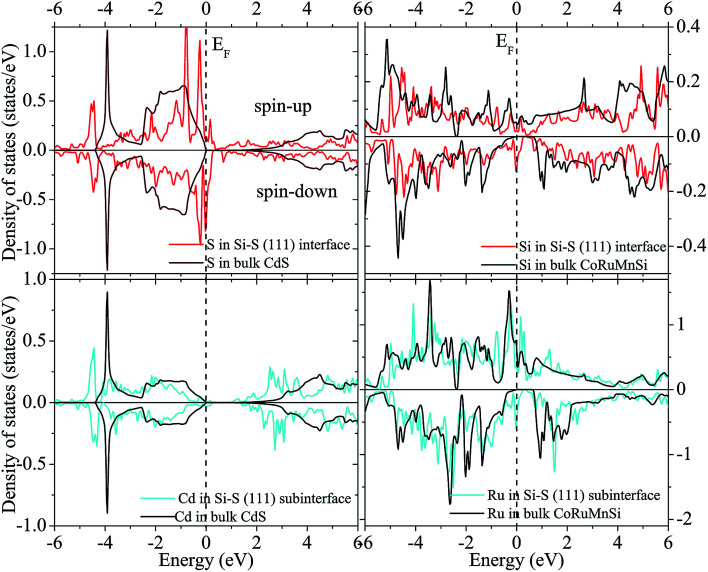
Similar to Fig. 12 for the case of the Si–S configuration.

Fig. 12 and 13 show the calculated spin-polarized partial DOS of the interface for the CoRuMnSi alloy. One can see that for the two interfacial structures of CoRuMnSi/CdS (111), the Fermi levels have intersections in both spin-up and spin-down states, which indicates that the half metallicity is ruined on the (111) interfaces for the CoRuMnSi alloy. Further, DOS of the interfaces as indicated in Fig. 12 and 13 is clearly different from the bulk. In addition, the atom-resolved DOS at the sub-interfaces and in the CoRuMnSi bulk are available for comparison. Unfortunately, the DOS of Si and Cd atoms at the Si–Cd (111) interface and Si and S atoms at the Si–S (111) interface show that the spin-up and spin-down channels have metallic characteristics. This means that the bulk half-metallicity of CoRuMnSi is destroyed at the Si–Cd and Si–S (111) interfaces because of the emergence of interfacial states inside the gap of the spin-down channel.

Finally, the calculated spin polarization and atomic spin magnetic moments of interface and sub-interface are listed in Table 3 with corresponding bulk values for comparison. For the first interfacial shape Si–S, the spin polarization is reduced down to 69% and 70% at the Si and S atoms, respectively, while spin polarizations of the sub-interface are 23% (Ru atom) and 0.2% (Cd atom). On the other hand, the Si–Cd shape has a spin polarization of about 16% and 7% at the Si and Cd atoms, respectively. Contrarily, the sub-interface spin polarizations are reduced to about 69% and 0.6% at the Ru and S atoms, respectively.

**Table 3:** The magnetic moments (in *μ*_B_) and spin polarization (*P*) at the CoRuMnSi/CdS (111) interface with Si–Cd and Si–S configurations, sub-interface (with *), the middle layer (in parenthesis), and in the bulk quaternary Heusler alloy CoRuMnSi

Interface structure	*M*				*P*			
	Si	Cd	S	Ru	Si	Cd	S	Ru
Si–Cd	−0.026(−0.027)	0.0065(0)	0.003*	0.014*	0.16	0.07	0.006*	0.69*
Si–S	−0.027(−0.028)	−0.0002*	−0.007(0)	−0.133*	0.69	0.023*	0.70	0.23*
Bulk	−0.003	0	0	−0.146	1.0	0	0	1.0

Obviously, atomic spin magnetic moments at the Si and S (111) interface (−0.027 and −0.007 *μ*_B_) increased compared to the bulk value (−0.003 and 0.0 *μ*_B_) due to the spin split strengthening of Si DOS as indicated in Fig. 12. Comparatively, the magnetic moments of the Si–Cd shape are −0.026 (Si) and −0.0065 *μ*_B_ (Cd). In addition, we perceive that the atomic spin magnetic moments are 0.014 *μ*_B_ at the sub-interface Ru for Si–Cd (111) interface, and −0.133 *μ*_B_ at sub-interface Ru for Si–S (111) interface. This means that the atomic spin magnetic moments at the (111) sub-interface became less compared to the bulk values because of the attenuating spin split as shown in Fig. 12 and 13.

## 4. Conclusion and summary

To sum up, we employed a first-principles investigation based on density functional theory to discuss the surfaces of CoRuMnSi (111), (110), and (001) and the interface of CoRuMnSi/CdS (111). It was found that the Si-terminated (111) surface maintains the HM character of the bulk Heusler compound CoRuMnSi. On the other hand, the Co, Ru, and Mn-terminated (111) surfaces, as well as both the MnSi- and CoRu-terminated (001) surfaces, destroy the bulk half-metallicity in CoRuMnSi, while the (110) surfaces have a nearly half metallicity. Besides, the half-metallicity of the bulk CoRuMnSi is wasted at the Si–Cd and Si–S shapes of the CoRuMnSi/CdS (111) interfaces. Furthermore, the spin magnetic moments and spin polarization values of surfaces and subsurfaces were also studied.

## Conflicts of interest

The authors declare that they have no conflicts of interest.

## Supplementary Material
